# Visual attention and cognitive workload using different laparoscopic box trainers and mixed-reality feedback

**DOI:** 10.1007/s00464-025-11881-4

**Published:** 2025-07-07

**Authors:** Aseel F. Khanfar, Sanaz Motamedi, Shawn D. Safford, Jason Moore, Jessica Menold, Scarlett Miller

**Affiliations:** 1 Department of Industrial Engineering, The Pennsylvania State University, University Park, PA USA; 2 Department of Industrial Engineering, Yarmouk University, Irbid, Jordan; 3 Division of Pediatric General and Thoracic Surgery, UPMC Children’s Hospital of Pittsburgh, 111 South Front Street, Harrisburg, PA 17101 USA; 4 Department of Mechanical Engineering, The Pennsylvania State University, University Park, PA USA

**Keywords:** Laparoscopic training, Visual attention, Mental workload, Eye-tracking metrics, Mixed-reality simulators, Pediatric laparoscopy

## Abstract

**Background:**

Laparoscopic techniques have revolutionized minimally invasive surgery (MIS) but remain visually and mentally demanding, especially in smaller operative sites (e.g., pediatric patients). Though traditional box trainers serve as the golden standard for laparoscopic simulation-based training (SBT), they are limited due to their lack of real-time feedback and objective assessments. Advancements in SBT using Visual Reality (VR) and Augmented/Mixed-Reality (AR/MR) can provide objective real-time evaluations. This study assessed the effects of mixed-reality feedback on trainees’ visual attention and mental workload during adult and pediatric laparoscopic SBT.

**Methods:**

Expert (*n* = 4) and novice surgeons (*n* = 24) were recruited to investigate the effects of various laparoscopic box trainers (e.g., adult and pediatric patients), as well as different feedback conditions in a MR setting, on trainees’ visual attention and perceived mental workload. Peg transfer task was chosen to evaluate novices’ ambidexterity and eye–hand coordination. Eye-tracking metrics (fixations, fixation durations, saccades, and saccade durations) and mental workload indicators (pupil diameter and NASA-TLX scores) were collected. K-means clustering was used to classify novices into proficiency groups based on these metrics.

**Results:**

Eye-tracking and mental workload metrics successfully differentiated two novice proficiency groups, significantly different from expert performance. Analysis revealed two key findings: novices demonstrated shorter fixation durations when using the pediatric trainer when compared to the adult trainer, and pupil diameter was lower for participants who started their trials with pediatric trainers (*p* = 0.016). However, the presence or absence of mixed-reality feedback did not significantly affect visual attention patterns or mental workload measures.

**Conclusions:**

Visual attention and mental workload metrics in mixed-reality environments effectively differentiated novice proficiency levels in laparoscopic box trainers. Our findings validate eye-tracking metrics for objective skill assessment in both adult and pediatric trainers, highlighting their potential for adaptive training programs and competency evaluation in surgical education.

**Graphical abstract:**

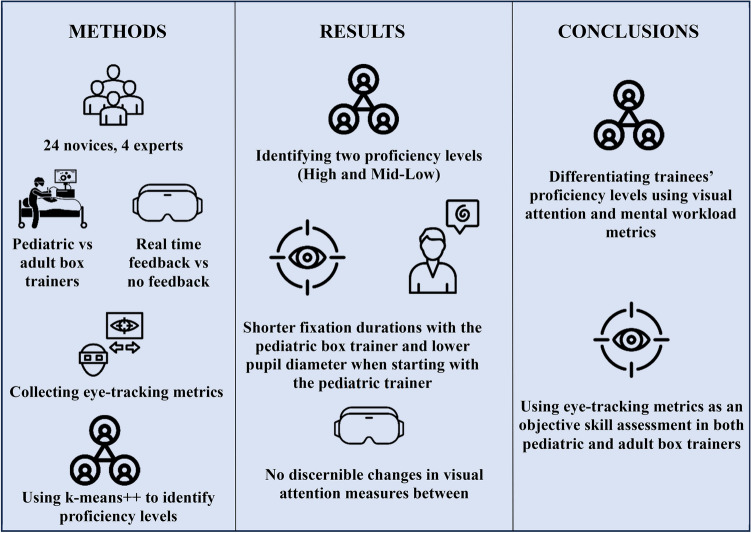

In the last three decades, laparoscopic techniques have transformed minimally invasive surgery (MIS) [1] with more than 15 million procedures performed annually worldwide [2]. These techniques provide significant benefits including reduced post-surgery pain, faster recovery times, shorter hospital stays, and smaller incisions compared to traditional open surgery [3, 4]. However, laparoscopic procedures present unique challenges: surgeons must rely heavily on visual cues to compensate for limited tactile sensation and restricted operative field visibility [5]. This makes laparoscopic surgery visually intensive and mentally demanding [6, 7]. The challenges are further amplified when performing the procedure on pediatric patients due to the smaller operative site and the tissue’s delicate nature [8]. Consequently, it is essential to provide comprehensive technical and cognitive skills training that allows medical professionals to develop proficiency in a low-stakes, low-stress environment before entering the operating room where error can lead to harm [9, 10].

Box trainers have become the gold standard of laparoscopic simulation-based training (SBT) used in fundamental laparoscopic skills (FLS), which is required to be completed by all surgical residents as part of their residency program [11]. While the effectiveness of the box trainers has been proven for laparoscopic basic skills training [12, 13], they have been criticized due to their lack of real-time feedback and objective assessment and the fact that they require trained proctors to be on-site for feedback and assessment [10, 14]. In addition, commonly used FLS box trainers only simulate adult patients, and the availability of validated pediatric box trainers for widespread training purposes is still limited [15].

Considering these deficiencies, more advanced simulators have been developed, including Visual Reality (VR) [18] and Augmented/Mixed-Reality (AR/MR) simulators [16–18]. VR simulators have been validated and shown to be effective in offering step-by-step, hands-on instructions for practice and real-time accurate assessment. Still, they often do not provide the haptic feedback necessary to enhance training performance and skill transfer [14, 19]. On the other hand, AR/MR simulators combine haptic input and real-time feedback properties, which can potentially be useful for laparoscopic training [14, 20]. While AR/MR simulators bridge the gap between VR simulators and box trainers and provide the potential for real use of laparoscopic instruments and automatic, objective assessment of performance, their utilization in surgical training has yet to be realized, and their effectiveness compared to other simulators remains unclear [21–23].

While the FLS scoring system has been the traditional method for evaluating laparoscopic skills and determining proficiency levels based on precision and accuracy metrics, its reliance on subjective assessment and lack of automatic feedback presents significant limitations [23–25]. Incorporating objective measures of trainees’ visual behavior and cognitive process could enhance the assessment of surgical competency [26, 27]. Eye movement has emerged as a measurable signal of skills acquisition, with prior work differentiating expertise levels based on trainees’ visual attention and measuring trainees’ perceived mental workload during different task difficulties in laparoscopic training [28–30]. Eye-tracking metrics such as fixations and saccades variations are used to measure the focus of the trainees’ visual attention [28, 31]. Additionally, pupil diameter is an important eye metric for measuring the perceived mental workload [32]. Previous research has demonstrated that pupil diameter decreased for expert surgeons who generally perceived lower mental workload and employed less effort than novices during laparoscopic training [33–35]. Additionally, it has been shown that pupil diameter increases with task difficulty, indicating greater cognitive demand [36].

In addition to the challenges associated with FLS, previous research has highlighted that training using the pediatric laparoscopic box trainer (PLS) presents greater difficulties for surgeons, particularly in terms of eye gaze metrics and mental workload [29, 32, 37]. Moreover, prior studies suggest that exposure to progressively more difficult training scenarios enhances skill development and accelerates proficiency [38].

Despite the challenges associated with FLS, eye-tracking metrics have proven effective in distinguishing between experts and novices based on their visual attention and perceived mental workload [39–41]. The progression from novice to expert is not binary—some novices can achieve intermediate proficiency levels relatively quickly after initial training [39]. Although these metrics offer potential benefits, little attention has been given to understanding how eye-tracking metrics can provide insights into the skill level of trainees, especially in the early stages of their training [39, 42]. Therefore, more research is needed to train and evaluate novice surgeons on pediatric simulators, due to the higher difficulty level of training. Additionally, investigating the effectiveness of AR/MR compared to other traditional simulators using robust evaluation tools such as eye-tracking is crucial. This study aimed to categorize trainees’ proficiency levels into groups/clusters based on eye-tracking metrics (e.g., visual attention) during FLS peg transfer task training on different box trainer anatomies (adult and custom-built pediatric box trainers), while also evaluating the impact of integrating an MR simulator to provide real-time feedback digitally during laparoscopic procedure training.

## Research questions

The main goal of this study was to assess visual attention and perceived mental workload in laparoscopic surgery training across different box trainer types (adult and pediatric) and mixed-reality feedback conditions. This was achieved by conducting an empirical study using the FLS peg transfer task to enhance novices’ ambidexterity and eye–hand coordination. Specifically, this work was designed to answer the following research questions:

### RQ1

Can measures of visual attention and perceived mental workload differentiate between proficiency levels of trainees?

It was hypothesized that different levels of proficiency would be identified among novices and that visual attention and perceived mental workload metrics would differ significantly between these levels. Specifically, fixations, fixation durations, saccades, and saccade durations as visual attention metrics were expected to be lower for higher proficiency levels compared to lower proficiency levels. Additionally, pupil diameter and NASA-TLX scores as mental workload metrics were hypothesized to be lower for high proficiency levels compared to lower proficiency levels.

### RQ2

Does the type (pediatric or adult) and order of training sessions influence visual attention and perceived mental workload?

We hypothesized that fixations, fixation durations, saccades, saccade durations, pupil diameter, and NASA-TLX scores would be higher in pediatric box trainers for novices during training and that visual attention and mental workload metrics would be lower for those who started their experimental trial with a pediatric trainer (a more challenging task) than those who began their trial with an adult box trainer.

### RQ3

Do different feedback conditions impact novices’ visual attention and perceived mental workload?

We hypothesized that novices’ eye metrics, such as fixations, fixation durations, saccades, and saccade durations, will increase across feedback conditions due to a feedback screen. On the other hand, we hypothesized that pupil diameter and NASA-TLX score would decrease across feedback conditions.

## Materials and methods

To answer these research questions, an experiment was conducted at the Simulation Center at Penn State Milton S. Hershey Medical Center (HMC). Detailed information about the experiment is discussed in the following sections:

### Participants

Twenty-eight participants (13 females and 15 males) were recruited for the IRB-approved study (STUDY0023069). Among the participants, twenty-four were surgical residents and medical students (novices) (*N*_*n*_ = 24, 12 females), and four were experts (*N*_*e*_ = 4, 1 female). As Buzink et al. suggested, we classified participants performed more than 50 laparoscopic surgeries as experts [46]. All novices had performed fewer than two laparoscopic surgeries in their career and had less than three years of experience. All participants had normal or corrected-to-normal vision, and five were left-handed. The racial and ethnic data of the participants can be found in Table 1.

**Table 1:** The racial and ethnic data of the participants

	Novice	Expert	Total
White	14	1	15
Asian	7	2	9
Black/African American	1	0	1
Hispanic/Latino	1	0	1
American Indian/Alaska Native	0	1	1
More than one race	1	0	1

### Procedure

At the beginning of the study, the experiment aims of the study were explained to participants, and informed consent was obtained. Afterward, participants were fitted with a Tobii Glasses 3 wearable eye tracker with a sampling rate of 50 Hz that was calibrated for each participant. Once fitted, participants were randomly assigned to one of four feedback conditions to explore the impact of mixed-reality feedback on training performance (see Table 2).

**Table 2:** Experts and novices’ distribution of different feedback conditions and two trainers (adult and pediatric) across different trial orders

Feedback conditions	Adult	Pediatric	Experts	Novices	
			Trial order 1	Trial order 1	Trial order 2
Feedback Condition 1 (FC1)	No feedback	No feedback	1	3	3
Feedback Condition 2 (FC2)	No feedback	Feedback	1	3	3
Feedback Condition 3 (FC3)	Feedback	No feedback	1	3	3
Feedback Condition 4 (FC4)	Feedback	Feedback	1	3	3

Trial order 1: participants started their initial trial using an adult box trainer and then a pediatric box trainer

Trial order 2: participants started their trials with a pediatric box trainer and then an adult box trainer

**Fig. 1:**
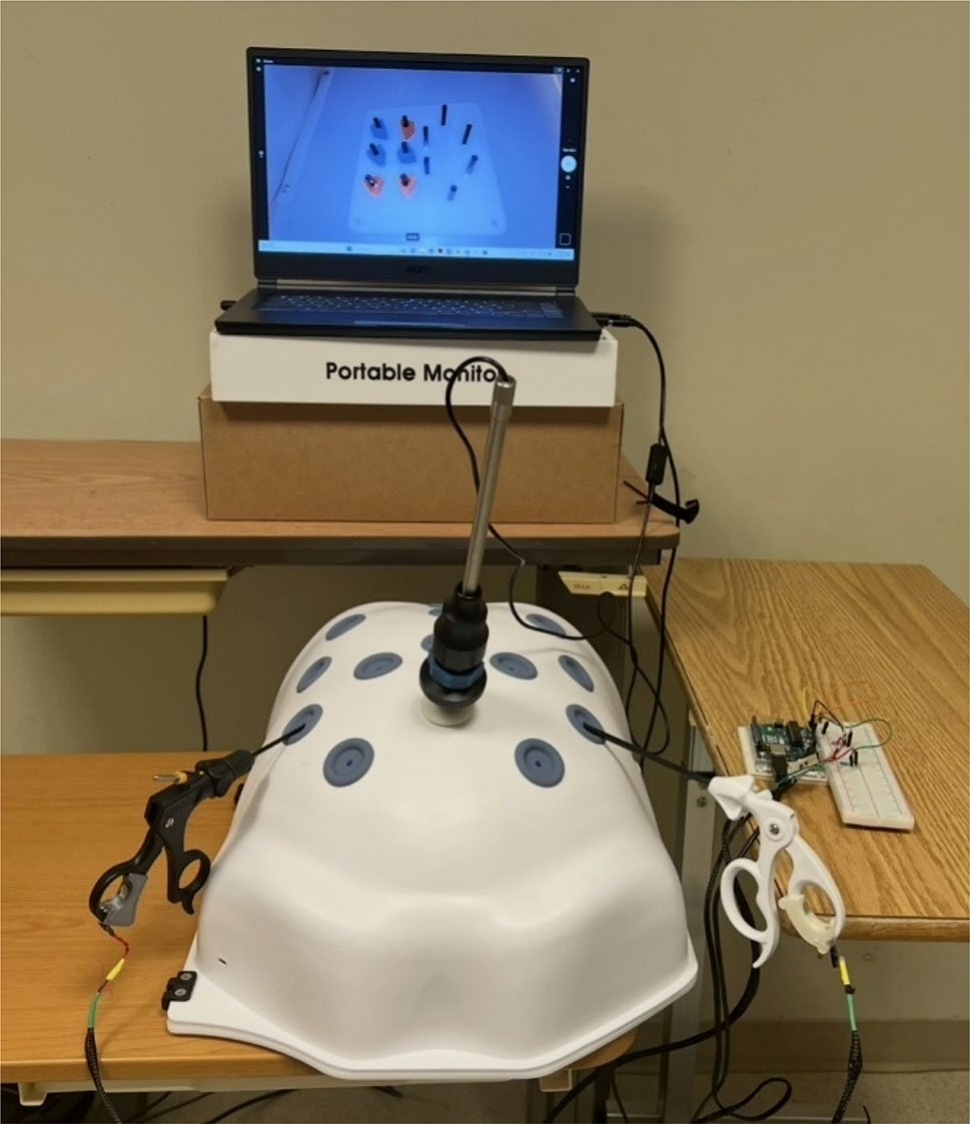
Experimental setup with the adult box trainer. Shows the experimental design setup as participants performed the peg transfer task. It includes a commercially available box trainer simulator connected to an optical camera, which displayed the inside of the box trainers on a 15-inch monitor

Participants were asked to complete the FLS peg transfer task using two box trainers:

1. A commercially available box trainer simulator from Medicinology and Co [47] that represents an adult patient/anatomy (see Fig. 1).
2. A custom-built pediatric trainer representing pediatric patient/anatomy (see Sect. “Feedback conditions” for more details).

To account for potential order effects, participants were assigned to one of two trial orders:

1. Trial order 1, in which participants started their initial trial using an adult box trainer and then a pediatric box trainer
2. Trial order 2, in which participants started their trials with a pediatric box trainer and then an adult box trainer.

Sixteen participants (*N*_*n*_ = 12 novices and *N*_*e*_ = 4 experts) were assigned to Trial order 1 and the rest of the participants (*N*_*n*_ = 12 novices) were assigned to Trial order 2. For both trainers, the simulators were connected to an optical camera that displayed the inside of the box trainers on a 15-inch monitor. Figure 1 represents the experimental setup using the adult box trainer.

**Fig. 2:**
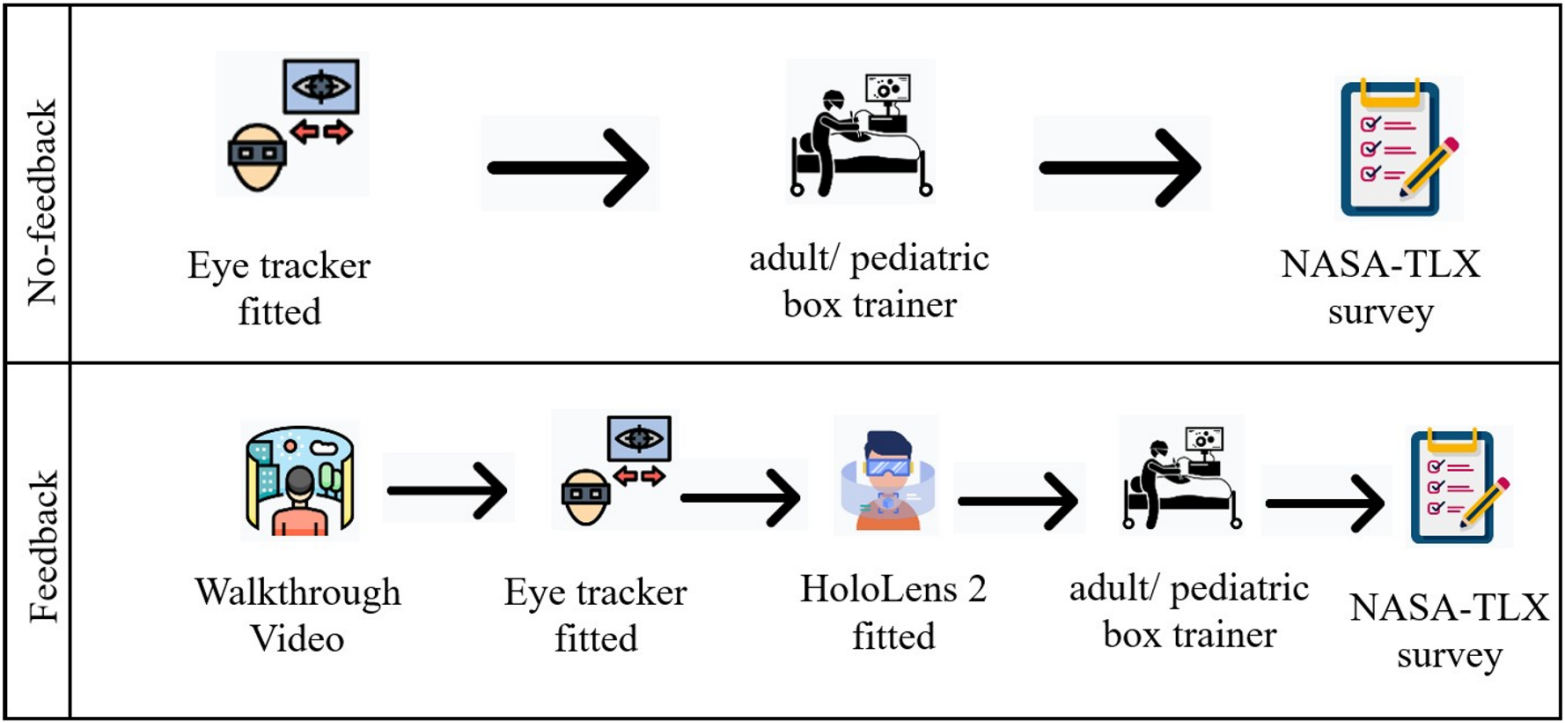
The overall experiment procedure across feedback conditions. Shows the overall procedure the experiment across different feedback conditions. All participants were fitted with an eye tracker for both conditions. In the feedback condition, participants were presented with a 32-s walkthrough video to familiarize themselves with the mixed-reality environment, then they were fitted with a HoloLens before start the procedure

**Fig. 3:**
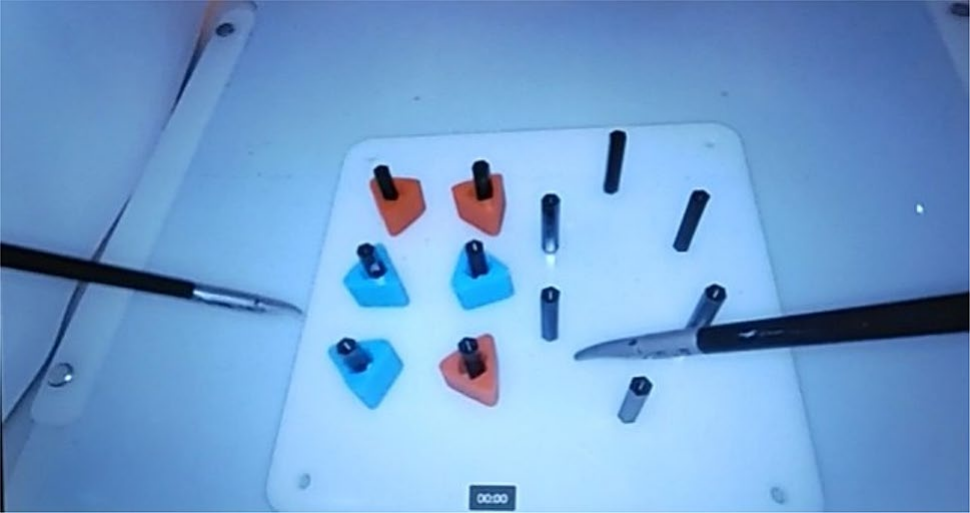
Peg transfer task. Shows the peg transfer task, where participants were asked to pick up the objects from the pegs with graspers in their non-dominant hand and transfer the objects to the grasper held by their dominant hand, without putting the peg down, in alignment with the FLS peg transfer

As shown in Fig. 2 based on the assigned feedback condition, participants were fitted with only an eye tracker in the no-feedback condition, while those in the feedback condition were presented first with a walkthrough video before being fitted with an eye tracker and the Microsoft HoloLens 2. Participants were then asked to complete the FLS peg transfer task according to FLS standards [48], where they were asked to pick up the object from the pegs with graspers in their non-dominant hand and transfer the object to the grasper held by their dominant hand, without putting the peg down, in alignment with the FLS peg transfer, see Fig. 3. Participants repeated this process for all six pegs. The color or sequence in which the six objects are transferred is unimportant. Participants completed only the first half of the task without reversing, as originally stated by the FLS manual, due to time constraints in the experiment. Following the successful completion of the task on the assigned box trainer, a NASA-TLX survey [49] was completed. Participants repeated this procedure on the other box trainer and completed a second NASA-TLX survey.

### Feedback conditions

The study implemented four distinct feedback configurations:

- FC1: No feedback for either trainer (control condition)
- FC2: Mixed-reality feedback for pediatric box trainer only
- FC3: Mixed-reality feedback for adult box trainer only
- FC4: Mixed-reality feedback for both trainers

Control Condition (No Feedback):

Participants completed the procedure using only standard training feedback mechanisms, such as visual inspection of object placement and movement.

Mixed-reality feedback condition:

The mixed-reality condition consisted of several components:

1. Initial orientation: Participants viewed a 32-s walkthrough video introducing the mixed-reality environment and its features.
2. Equipment setup: Participants were fitted with a Microsoft HoloLens 2 mixed-reality headset with integrated eye-tracking capability (30 Hz sampling rate).
3. Calibration: Individual calibration ensured accurate eye-tracking for each participant.
4. Real-time feedback: The system displayed force measurements and time metrics during task execution.

### Apparatus

**Fig. 4:**
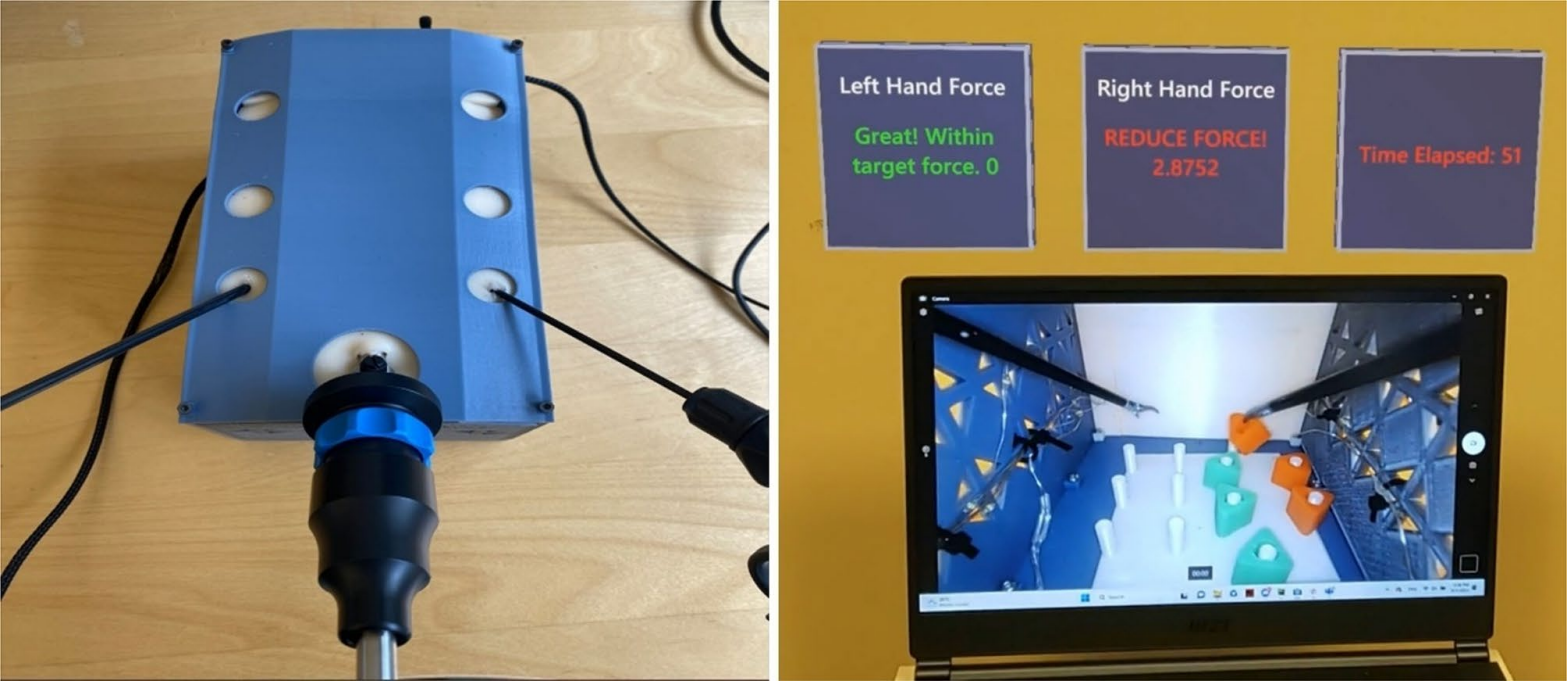
Customized PLS box trainer (left), Mixed-reality feedback screen integrated with PLS box trainer (right). Shows a low-fidelity, customized pediatric box trainer (PLS) in the left image. The PLS box trainer is designed for pediatric laparoscopy training and featuring a 3D-printable design. The right image shows the MR feedback screen, where metrics change from green to red, indicating high pressures being applied on left and right graspers by participants’ hands and the task taking more than 48 s to perform (proficiency time)

The mixed-reality simulator, which has been recently developed, was connected to the adult FLS simulator with internal dimensions 455 × 395 × 220 mm and a low-fidelity customized pediatric box trainer (PLS), see Fig. 4. The PLS box trainer, designed for pediatric laparoscopy training and featuring a 3D-printable design, followed the baseball diamond concept [50] to strategically position ports for effective instrument triangulation. Compact in size with dimensions of (140 × 230 × 126 mm), it prioritized user-friendly interactions. The inclusion of a replaceable Brrnoo training suture pad simplified maintenance. The 1920 × 1080 resolution camera ensures visual clarity, and the design allows natural light entry. Ohmite FSR05 force-sensing resistors were attached to 3 mm “Laparo Scopy Boxx” pediatric laparoscopic needle holders. To synchronize and update the force values, these resistors were connected to an Arduino board through Python code. The feedback screen metrics change from green to red, signaling high pressures being applied on left and right graspers by participants’ hands and the task taking more than 48 s to perform (proficiency time) [51]. Force thresholds were set based on the sensitivity of the force sensors.

### Metrics

Various eye-tracking metrics were used during the study to evaluate visual attention and perceived mental workload. The eye-tracking metrics used [52, 53] are summarized in Table 3.

**Table 3:** Eye-tracking metrics used in the study

Eye-tracking Metrics	Description	Unit
Total fixation durations	The sum of durations of all points at which eyes are stationary at the exact location	Seconds
A total number of fixations	Total number of all points at which the eye is stationary at the exact location	Count
Total saccade durations	The sum of durations of rapid eye movements between two fixations	Seconds
A total number of saccades	Total number of all points of rapid eye movements between two fixations	Count
Dwell Time	The sum of fixation and saccade durations	Seconds
Pupil diameter	Average changes in pupillary size	Millimeter

In addition to pupil diameter, which was used to evaluate participants perceived mental workload during the training sessions, a subjective mental workload assessment was also used. NASA-TLX scale is a robust subjective assessment of mental workload and has been extensively used in surgical and medical domains [54]. It consists of six dimensions: mental demand, physical demand, temporal demand, performance, effort, and frustration. Each participant was asked to rate the perceived mental workload for each dimension on a scale of 20 at baseline (before the training) and after each training session. After the two sessions were completed, a 15-paired comparison matrix was filled to calculate the weight of each dimension. Each dimension’s workload score (ranging from 0 to 100) was calculated by multiplying the weight (ranging from 0 to 5) by the rating score (ranging from 0 to 20) for each dimension. A total NASA-TLX score was then determined by summing each dimension’s final score and dividing by 15 (ranging from 0 to 20).

### Data analysis

Before extracting eye-tracking metrics, gaze sample percentages were reviewed for all recordings during each training session to check the percentage of eye gaze data captured from glasses when the participants looked directly through them, not outside the glasses’ boundary. The gaze sample percentages for all recordings were at least 82%, except for two trials with 43% (pediatric trial) and 44% (adult trial) gaze sample percentages, which were excluded from the analysis. This led to a total of 54 trials in the analysis.

The Tobii Velocity-Threshold Identification (I-VT) fixation filter was used to extract eye-tracking metrics in Tobii Pro Lab software [55]. Thirty degrees/sec is the default threshold velocity, where eye movement with a more than 30 degrees/sec velocity is classified as saccade, and less than 30 degrees/sec is classified as fixation. However, this value was tested on stationary eye trackers. For wearable eye trackers (e.g., Tobii Glasses 3), 70–110 degrees/sec was suggested for recordings performed under dynamic situations [56, 57]. Hence, a 70-degree/sec velocity threshold was chosen for this study’s analysis, as previous research has shown its effectiveness in detecting saccades and fixations without losing precision [58, 59].

All statistical analyses were analyzed using IBM SPSS (V. 29.0) at a significant level of 0.05. Data are mean ± standard deviations unless otherwise stated. For the entire study in this paper, if outliers were present, tests were conducted with and without them. If the outcomes remained consistent, the outliers were not excluded. Otherwise, they would be removed. A one-way Welch ANOVA was conducted if the homogeneity of variances was violated.

As an initial step, a two-way ANOVA was run to assess the effect of (feedback conditions-trainer type), (feedback conditions-trial order), and (trainer type-trial order) on visual attention and mental workload metrics. All metrics had no statistically significant two-way interactions between (feedback conditions-trainer type) and (feedback conditions-trial order). Consequently, the subsequent analysis in the paper was performed independently for the feedback condition factor.

## Results

This section presents our findings with reference to the research questions.

### RQ1

Can measures of visual attention and perceived mental workload differentiate between proficiency levels of trainees?

K-means++ clustering [60] was used to identify different levels of task proficiency. Three groups were determined by plotting the within-cluster sum of squares against the number of clusters: Group 1 (23 trials), Group 2 (17 trials), and Group 3 (6 trials). The expert group (8 trials) was not included in the clustering analysis to ensure the accuracy of the clustering method. See Table 4 for descriptive analysis.

**Table 4:** Descriptive analysis of the three suggested groups and the expert group

Metrics groups		Time	Fixation number	Fixation duration	Saccade number	Saccade duration	Pupil diameter	NASA-TLX
**Expert**	**Mean**	**69.00**	**28.25**	**67.92**	**33.25**	**0.87**	**3.33**	**9.54**
	**Median**	**71.50**	**23.50**	**71.05**	**26.00**	**0.63**	**3.37**	**3.67**
	**Std**	**18.12**	**25.70**	**17.86**	**34.09**	**0.81**	**0.35**	**3.63**
Group 1	Mean	170.04	89.52	164.88	122.39	3.94	3.90	10.17
	Median	160.00	65.00	157.15	79.00	2.66	3.82	10.26
	Std	59.05	73.19	58.71	103.12	3.34	0.48	2.86
Group 2	Mean	320.64	260.47	301.39	401.17	11.99	4.14	10.57
	Median	341.00	221.00	321.34	405.00	12.14	4.21	10.86
	Std	88.95	78.49	93.20	143.78	3.77	0.69	2.58
Group 3	Mean	367.66	764.33	290.93	1319.16	40.07	4.85	11.47
	Median	324.50	662.00	258.00	1178.50	36.35	5.09	12.70
	Std	156.59	315.92	146.95	479.51	14.03	0.76	3.32

Statistical analyses for experts are highlighted in bold

**Fig. 5:**
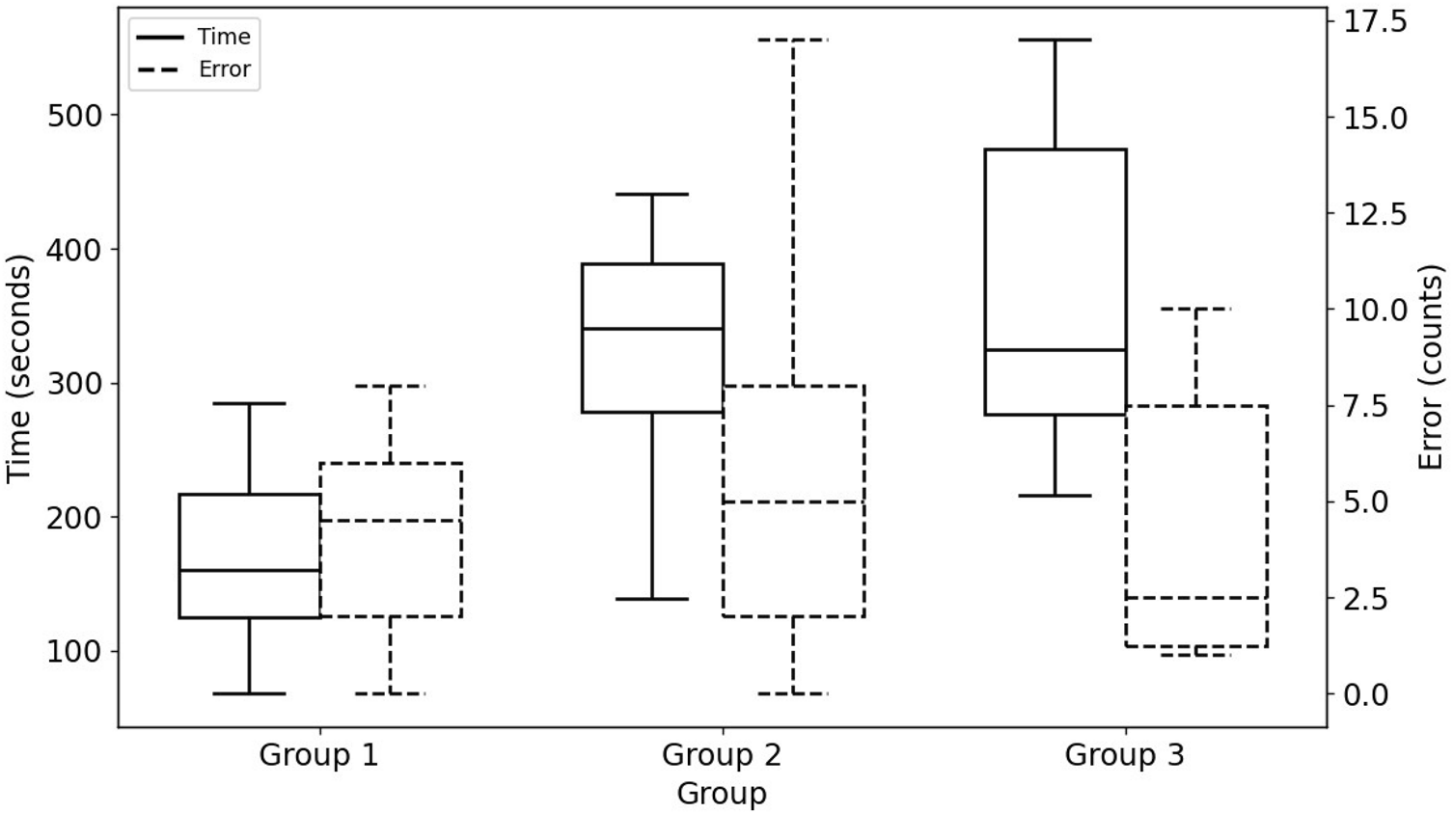
Number of errors committed and completion times between the three suggested groups. Shows boxplots for the number of errors committed (count) and completion times (seconds), which were used to identify the proficiency level for each defined group (cluster). The groups were identified using kmeans++ clustering based on participants’ visual attention and mental workload metrics

One-way Welch ANOVA on errors and completion times for each defined group were used to identify the proficiency level, see Fig. 5. The differences between the suggested groups were not statistically significant for the number of errors committed, Welch’s F(2, 12.092) = 1.435, *p* = 0.276. On the other hand, the completion times were statistically significantly different between the groups, Welch’s F(2, 11.798) = 21.122, *p* < 0.001. Games–Howell post hoc analysis revealed that the mean increase from Group 1 to Group 2 (150.61, 95% CI [88.89, 212.31]) s was statistically significant (*p* < 0.001), as well as the increase from Group 1 to Group 3 (197.26, 95% CI [16.05, 379.19], *p* = 0.036) s. There were no differences in completion times between Group 2 and Group 3. These results suggest that the clustering algorithm of visual attention and mental workload metrics classified novices into three groups (e.g., Groups 1, 2, and 3). However, the differences in completion times led to the identification of two proficiency levels: High (Group 1) and Mid-Low (Groups 2 and 3).

**Fig. 6:**
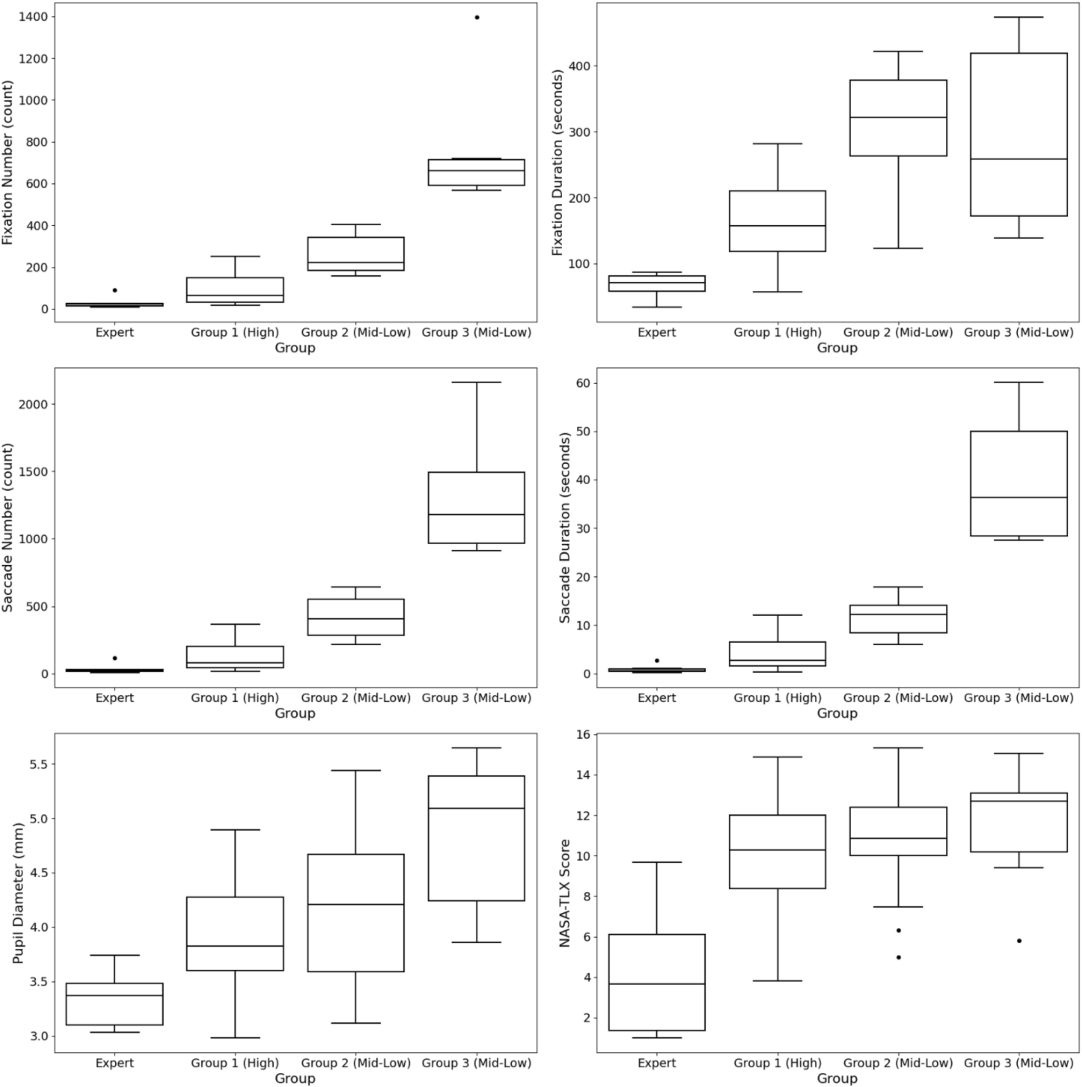
Box plots comparing visual attention and mental workload metrics between experts and the three suggested groups. Shows box plots comparing visual attention and mental workload metrics across three suggested groups and experts. All metrics showed a statistically significant difference between the suggested groups and the expert group

A one-way Welch ANOVA was conducted to determine whether the visual attention and mental workload metrics differed among the groups. All metrics showed a statistically significant difference between the groups (*p* < 0.001, see Fig. 6). Pairwise comparisons were conducted using Games–Howell analysis for fixation durations, pupil diameter, and NASA-TLAX metrics and Dunn [61]‘s procedure for fixations, saccades, and saccade durations as they failed the normality assumption (see Table 5). These analyses showed a statistically significant difference from the expert group across all visual attention and mental workload metrics (*p* < 0.05), with the expert group showing the lowest values. This was followed by Group 1, Group 2, and Group 3. However, for Group 1, which represented a high proficiency level, there was no statistically significant difference from the expert group in the metrics of fixations, saccades, and saccade duration metrics. A one-way Welch ANOVA on NASA-TLX dimensions showed significant differences in temporal demand, performance, and frustration between novices and experts (*p* ≤ 0.001). Games–Howell post hoc analysis revealed higher temporal demand, performance, and frustration for novices, with significant differences from experts (*p* < 0.05).

**Table 5:** Pairwise comparison between the three suggested groups of proficiency levels and an expert group using Games–Howell analysis

Metrics groups	Mean difference	Games–Howell analysis			Median difference	Dunn [61]’s procedure		
		Fixation duration	Pupil diameter	NASA-TLX		Fixations	Saccades	Saccade durations
Expert, 1	p-value	** < 0.001**	**0.002**	**0.003**	p-adjusted	0.469	0.549	0.368
	Difference	− 96.95	− 0.57	− 6.05	Difference	− 41.50	− 53.00	− 2.03
Expert, 2	p-value	** < 0.001**	**0.002**	**0.002**	p-adjusted	**0.00**	**0**	**0**
	Difference	− 233.47	− 0.81	− 6.45	Difference	− 197.50	− 379.00	− 11.51
Expert, 3	p-value	**0.048**	**0.014**	**0.008**	p-adjusted	**0.00**	**0**	**0**
	Difference	− 223.00	− 1.52	− 7.36	Difference	− 638.50	− 1152.50	− 35.72
1, 2	p-value	** < 0.001**	0.61	0.967	p-adjusted	**0.002**	**0.001**	**0.001**
	Difference	− 136.51	− 0.24	− 0.39	Difference	− 156.00	− 326.00	− 9.48
1, 3	p-value	0.275	0.095	0.816	p-adjusted	**0.00**	**0**	**0.00**
	Difference	− 126.04	0.95	− 1.30	Difference	− 597.00	− 1099.50	− 33.69
2,3	p-value	0.998	0.263	0.927	p-adjusted	0.447	0.52	0.487
	Difference	10.46	− 0.71	− 0.91	Difference	− 441.00	− 773.50	− 24.21

Significant *p* values are highlighted in bold

Pairwise comparisons between Group 1(High)-Group 2 (Mid-Low) and Group 1 (High)-Group 3 (Mid-Low) showed a statistically significant difference (*p* < 0.05) in fixation numbers, saccade numbers, and saccade durations, where all metrics significantly increased from Group 1 to Group 2, and from Group 1 to Group 3. No statistical significance was found between Group 2 and Group 3 across these metrics. For fixation durations, statistically significant results showed between Group 1 and Group 2 (*p* < 0.001). However, no statistical significance was found between Group 1 and Group 3 (*p* = 0.275), and Group 2 and Group 3 (*p* = 0.998). Pupil diameter and NASA-TLX were not statistically significantly different between the suggested groups (*p* > 0.05).

These results support our hypothesis that the groups obtained from the clustering analysis represent multiple proficiency levels based on visual attention. No differences were found in perceived mental workload metrics.

### RQ2

Does the type (pediatric or adult) and order of training sessions influence visual attention and perceived mental workload?

To evaluate the effect of trainer type and training sessions order on visual attention and mental workload metrics, two-way mixed ANOVA was run. A statistically significant interaction was found between the task type and the trial order on fixation duration, F(1, 20) = 6.117, *p* = 0.022, partial *η*^2^ = 0.234. Participants who started with the pediatric box trainer had significantly longer fixation durations than those who started with the adult box trainer (mean difference = 123.881, standard error = 41.717 s, *p* = 0.007). Additionally, task type had a statistically significant effect for those who started with an adult box trainer, F(1, 11) = 23.153, *p* < 0.001, partial *η*^2^ = 0.678, with longer fixation durations in the adult box trainer (mean difference = 112.62, standard error = 23.405 s, *p* < 0.001). No significant interaction was found for other metrics.

**Fig. 7:**
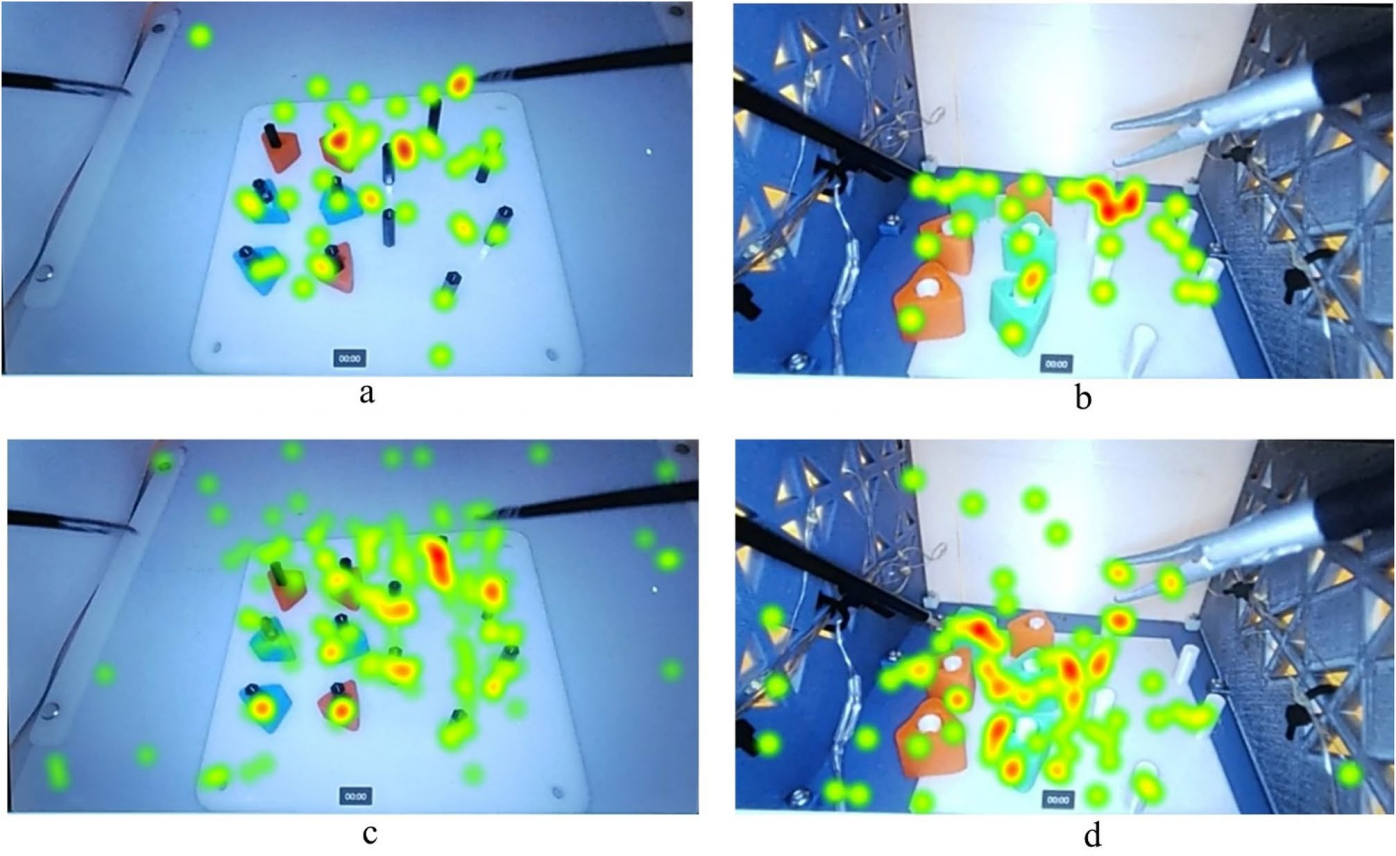
Heatmaps for **a** trial order 1 at adult trainer (Novice 1), **b** trial order 1 at pediatric trainer (Novice 1), **c** trial order 2 at adult trainer (Novice 2), and **d** trial order 2 at pediatric box trainer (Novice). Shows heatmaps to distinguish fixation counts and fixation durations between participants across different trial orders and trainer types. The heatmaps clearly show that fixation durations for novices were shorter in pediatric box trainer compared to adult box trainer

Follow-up analysis was performed to study the main effect of the task type and trial order across all metrics. The main effect of the task type showed a statistically significant difference in fixation duration, F(1, 20) = 15.554, *p* < 0.001, partial *η*^2^ = 0.437, with longer fixation durations in the adult box trainer (mean difference = 69.231, standard error = 17.55 s, *p* < 0.001). The main effect of the trial order showed a statistically significant difference in pupil diameter, F(1, 20) = 6.85, *p* = 0.016, partial *η*^2^ = 0.255, with higher pupil diameters for participants who started with the pediatric box trainer (mean difference = 0.65, standard error = 0.248 mm, *p* = 0.016). Figure 7 visualizes heat maps for two novices under both trial orders and the same feedback condition (FC1).

These results do not show that pediatric box trainer would be more challenging for participants using the visual attention and mental workload metrics. In fact, some metrics showed the opposite; for example, fixation durations for novices were lower in pediatric box trainer. The results also refute our hypothesis that visual attention and mental workload metrics would be lower for participants who started their trials with pediatric box trainers.

### RQ3

Do different feedback conditions impact novices’ visual attention and perceived mental workload?

**Fig. 8:**
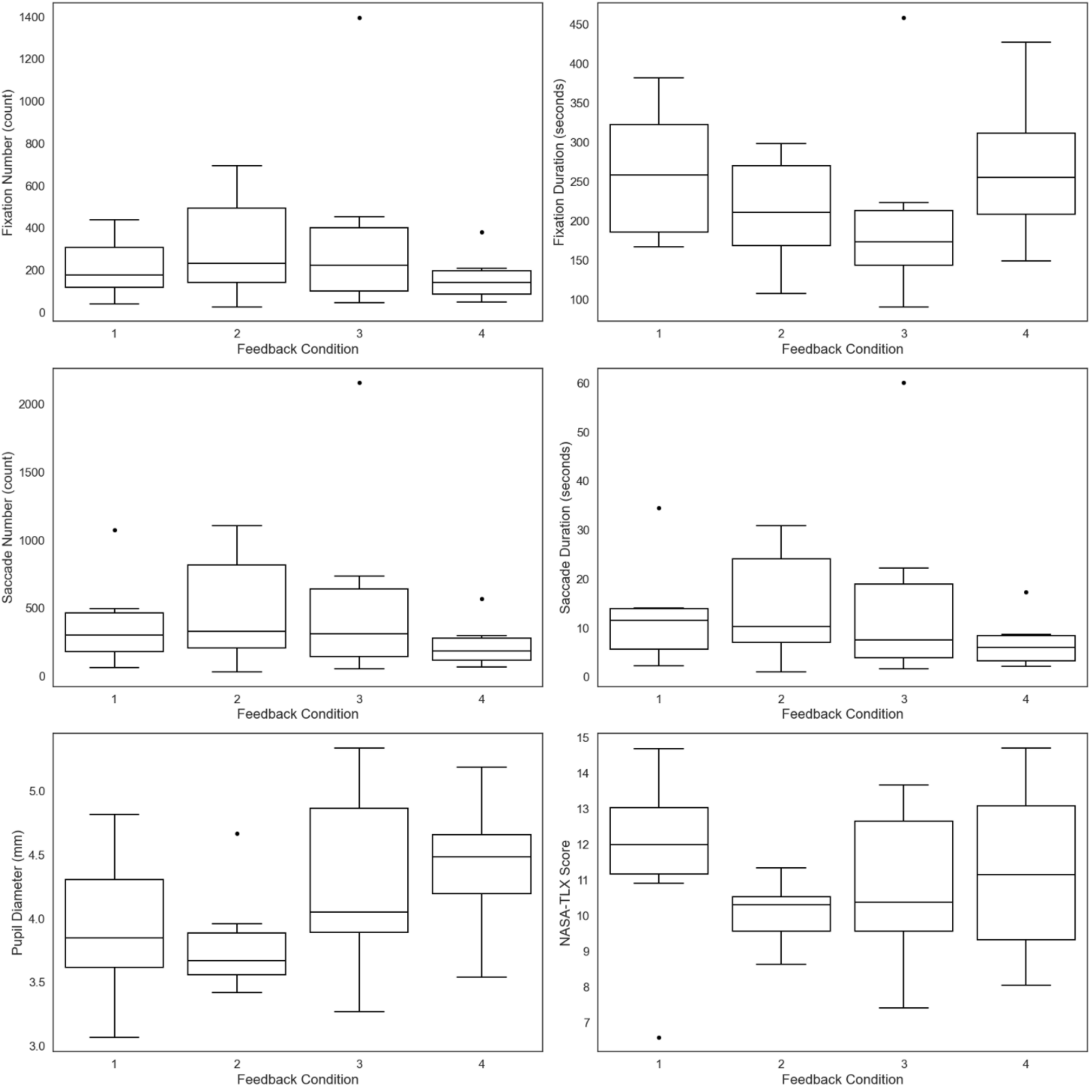
Box plots showing visual attention and mental workload metrics among novices under different feedback conditions. Shows box plots for visual attention and mental workload metrics across different feedback conditions. It indicates that the presence of a feedback screen (FC2, FC3, FC4) during training did not affect novices’ gaze behavior compared to no-feedback conditions (FC1)

To study the impact of feedback conditions across all metrics, one-way ANOVAs were conducted. Participants were classified into four groups: no feedback in both trainers (FC1) (*n* = 6), feedback in the pediatric trainer (FC2) (*n* = 6), feedback in the adult trainer (FC3) (*n* = 6), and feedback in both trainers (FC4) (*n* = 6). Metrics for both box trainers were averaged to assess each condition, as there is no interaction effect between feedback conditions and task type. Fixations, saccades, and saccade durations were the lowest in the FC4 condition (166.66 ± 119.53), (234.16 ± 182.71), and (7.23 ± 5.6) s, respectively, where participants received feedback on both box trainer tasks, as shown in Fig. 8. On the other hand, pupil diameter was the highest at FC4 (4.43 ± 0.55) mm, compared to FC1 (3.93 ± 0.62) mm, FC2 (3.82 ± 0.45) mm, and FC3 (4.27 ± 0.79) mm. Fixation durations were the highest at FC1 (261.93 ± 89.64) s and FC4 (268.41 ± 98.59) s, compared to FC2 (211.95 ± 73.31) s and FC3 (209.29 ± 130.17) s. However, the differences between these feedback conditions were not statistically significant for all metrics (*p* > 0.05). In addition, the overall NASA-TLX scores were the highest at FC1 (10.86 ± 2.95), C4 (10.72 ± 1.72), and FC2 (10.54 ± 1.62) compared to FC3 (9.93 ± 2.26). However, the differences were not statistically significant.

**Fig. 9:**
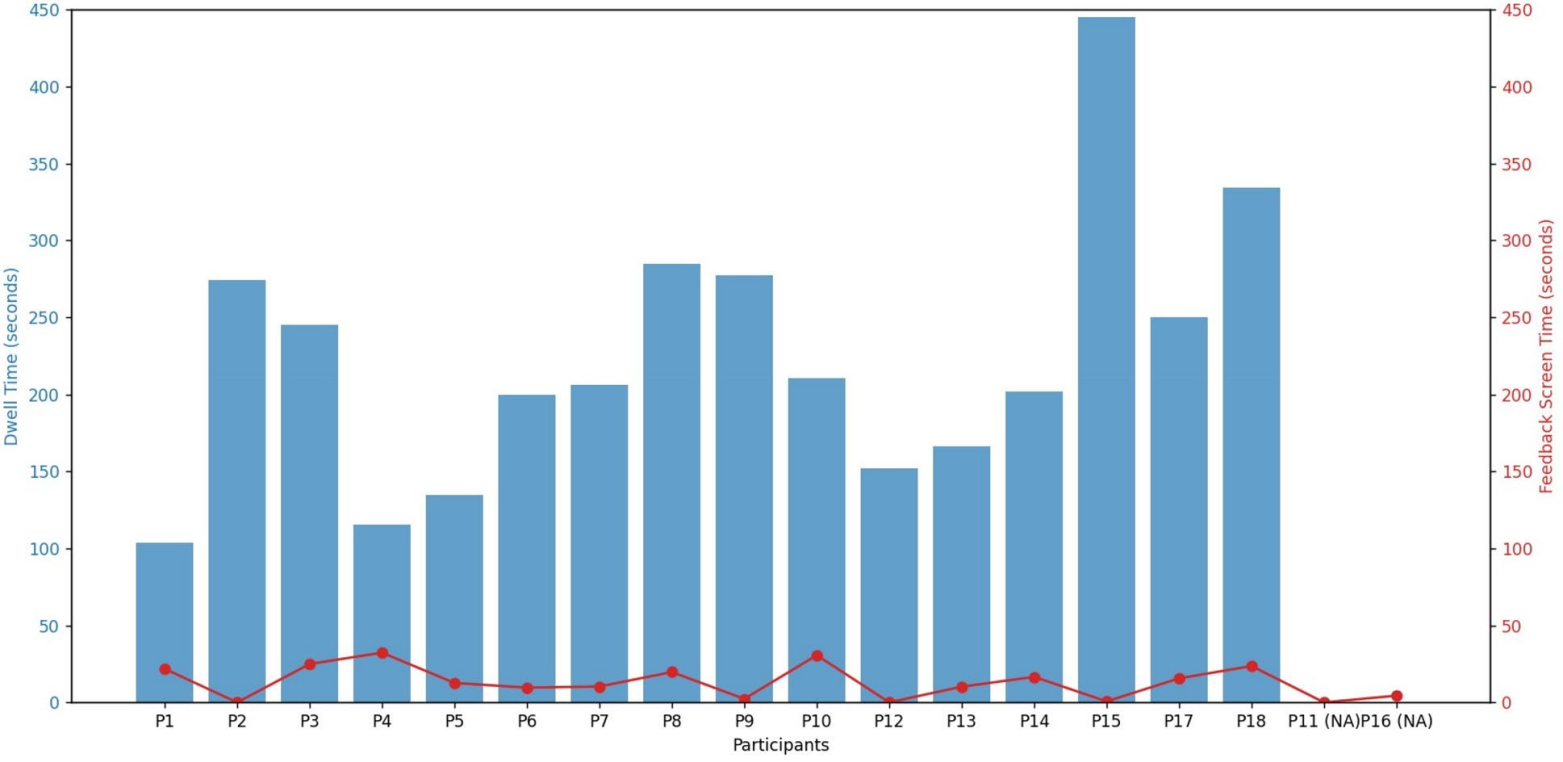
Comparison of novices’ dwell time with their total time on the feedback screen. Shows a dual axis figure to compare novices’ dwell time with their total time spent on the feedback screen to investigate whether novices paid attention to the feedback screen in the mixed-reality environment. The left X-axis represents dwell time, the right X-axis represents the time the participants spent looking at the feedback screen, and the Y-axis represents the individual participants

The results showed that the feedback screen did not affect novices’ gaze behavior compared to no-feedback conditions. To assess their attention to the feedback screen, we compared dwell time with the total time they spent looking into the screen (See Fig. 9). Dwell time is an eye-tracking metric that represents the total visual attention time during the training, and it is measured by summing all fixation and saccade durations [62]. On average, novices spent 8.04% of their time interacting with or focusing on the feedback screen during the training session. These results suggest that novices are potentially paying attention to the feedback screen during training.

## Discussion

The main goal of this work was to assess visual attention and perceived mental workload in laparoscopic surgery training across different box trainer types (adult and pediatric) and mixed-reality feedback conditions. The results for the **first research question** align with our hypothesis that visual attention and perceived mental workload metrics are effective indicators for classifying novice trainees into multiple proficiency levels in the FLS peg transfer task. Based on the clustering analysis, novices’ visual attention and mental workload were classified into three groups, while the differences in completion times indicated two proficiency levels: High (Group 1) and Mid-Low (Groups 2 and 3). The three groups were compared to the expert group, and there were significant differences across all visual attention and mental workload.

In terms of visual attention metrics, fixation durations were lower for the expert group compared to the three novice groups. Additionally, fixations were lower for the expert group compared to Groups 2 and 3, which represented Mid-Low proficiency levels, supporting previous work in epidural block training and laparoscopy surgery that showed fixation numbers and fixation durations were lower for experts than novices [35, 64]. Similar results were found for saccades and saccade durations, where the expert group had significantly lower values than Groups 2 and 3, indicating that novices have a high level of indecisiveness and uncertainty due to a lack of experience, as stated by another research [43]. There were no statistical differences between the expert group and Group 1 in terms of fixations, saccades, and saccade durations. Group 1 was statistically significantly lower than Group 2 and Group 3 across all visual attention metrics, except the fixation durations between Groups 1 and 3. These results indicate that Group 1, which had the highest proficiency level, showed similar visual behavior to the expert group. No statistical differences were found between Group 2 and Group 3 across all visual attention metrics, as they had the same proficiency level. However, the visual attention values for Group 2 were lower than Group 3, potentially indicating that Group 2 had more proficiency than Group 3. More research is needed to understand the behavior and proficiency levels of Group 2 and Group 3 [42, 65].

Pupil diameter and NASA-TLX scores as indicators of perceived mental workload during the task also align with our expectations and findings from previous research in endoscopic skull training and laparoscopic suturing tasks [33, 40, 44]. In these studies, lower NASA-TLX and pupil diameter were associated with higher expertise levels. In our work, pupil diameter and total NASA-TLX score were significantly higher for novices than the expert group, while no differences were found between the suggested groups. This suggests that novices allocate more attention and cognitive effort into completing the task, and regardless of their proficiency levels, they all showed high levels of mental workload. In addition, the results showed a statistically significant difference between experts and the suggested groups across temporal demand, performance, and frustration subscales. This can be explained by the less skilled novices who may find the peg transfer task more time-consuming and less effective, making it longer, harder to do well, and more frustrating.

The results for the **second research question** showed a statistically significant interaction between the task type and the trial order on the fixation duration metric for novices. Our hypothesis suggested that PLS box trainer would be more challenging and visual attention metrics would be higher for PLS box trainers, but the results refuted this hypothesis. Fixation duration was lower in the PLS box trainer than in the FLS box trainer. This may indicate that working on a larger operating site requires more extensive visual scanning and longer fixation durations to work in the entire area. The results also showed that participants who started their trials with the adult trainer and then switched to the pediatric trainer had less fixation duration at the pediatric trainer level. This finding aligns with the previous research in which more challenging training improved skill levels [38]. The effect of the trial order on the perceived mental workload metrics also supported our findings; the results showed that pupil diameter was lower when participants started their trial with an adult trainer. Starting the training with the more extensive visual scanning task required by an adult trainer may better prepare participants for managing their visual attention of a pediatric trainer.

The results for the **last research question** indicated that even with the existing feedback screen in mixed-reality, novices’ visual attention metrics for the whole training session were not statistically significant compared to no-feedback condition [66]. While insignificant, the total NASA-TLX score and pupil diameter were approximately high at the FC4 condition, where participants received both tasks with MR feedback. Consequently, the presence of a feedback screen may induce a higher level of cognitive load as it requires participants to shift their attention between the task and the feedback screen. This indicates that the virtual environment may require further development to be suitable for training sessions, as previous research stated that a well-designed virtual environment could effectively decrease mental workload [45, 67, 68]. Although it was hypothesized that visual attention metrics would be higher in feedback conditions, the sample size for each condition was relatively small (6 participants in each condition) and did not give a comprehensive understanding of the effect of feedback on visual attention patterns for participants. However, an additional analysis demonstrated that novices spent 8.04% of their time looking at the feedback screen during the feedback conditions. Compared to the previous research [63], which found that novices barely (approximately 0% of their time) looked at the vital monitor in laparoscopic training, the participants in this study seemed to pay attention to the feedback screen. The reason for the insignificant differences between the feedback and no-feedback conditions may be indicated by participants splitting their attention between the feedback screen and the task itself.

## Limitations and future work

Although the research showed promising findings in the medical training domain, the study has several limitations. This study was conducted at a large academic institution, and participation relied on the availability of residents and experts, impacting the sample size. Although eye-tracking metrics used in this study effectively differentiated expertise levels during the training sessions, these metrics, called AOI-independent eye-tracking metrics, were collected over the entire training sessions. In order to provide a comprehensive evaluation of how trainees direct their gaze and whether novices look on the same areas as experts, AOI-dependent metrics related to specific areas of interest (AOIs), e.g., fixation durations on AOIs, should be used [65]. However, extracting AOI-dependent gaze metrics from AOIs under dynamic scenes like medical training is more complex than static scenes. Future work should consider developing machine learning and computer vision algorithms to automatically extract dependent eye metrics on dynamic AOIs to investigate participants’ training behavior at various expertise levels. Due to time constraints, participants in this work completed only the first half of the peg transfer task. Additionally, all experts started their trials with the adult box trainer before switching to the pediatric box trainer. Future work should consider a complete FLS peg transfer task procedure and include more experts across different trial orders to enhance the robustness of the findings. Finally, to ensure participants’ focus on the feedback screen, other feedback formats, such as verbal and haptic feedback, can be explored, and their task performance can be evaluated. Additionally, follow-up interviews and post-surveys can be included in these studies to provide insights into participants’ perceptions of these feedback conditions.

## Conclusions

This research aimed to assess expertise levels and investigate if different laparoscopic box trainers and feedback could affect trainees’ visual attention and mental workload using eye-tracking metrics as an objective assessment tool and NASA-TLX metric as a subjective tool. The first main findings were that the expertise level could be distinguished by eye-tracking metrics such as fixations, fixation durations, saccades, and saccade durations during the training sessions. Also, pupil diameter was used as an eye-tracking metric, and NASA-TLX was used as a subjective mental workload assessment, differentiating the expert group from other novice proficiency levels. Second, we found that novices improved their visual attention patterns in the PLS box trainers compared to the FLS box trainers. Finally, we found that novices potentially pay attention to the feedback screen in a mixed-reality environment during the training. Therefore, we can conclude that eye-tracking metrics can effectively capture the difference in visual attention and perceived mental workload between trainees during the FLS peg transfer task and that differences in gaze patterns can also distinguish between PLS and custom-built FLS box trainers.
